# Special Issue: Dental Implant Materials 2019

**DOI:** 10.3390/ma13245790

**Published:** 2020-12-18

**Authors:** In-Sung Luke Yeo

**Affiliations:** Department of Prosthodontics, School of Dentistry and Dental Research Institute, Seoul National University, 101, Daehak-Ro, Jongro-Gu, Seoul 03080, Korea; pros53@snu.ac.kr; Tel.: +82-2-2072-2661

The Special Issue, “Dental Implant Materials 2019”, has tried to introduce recent developments in material science and implant dentistry with biologic and clinical aspects. Biocompatibility, design and surface characteristics of implant materials are very important in the long-term clinical service of dental implants. Ten original research articles and three review articles in this issue are considered to show well the significance of such factors from the clinical point of view.

Hard tissue response to implant surface is one of main fields many researchers are involved in. Surface modification technologies for implants have begun to be applied to titanium at the micro-level for about four decades. Currently, implant surfaces are being topographically and chemically modified at the micro- and nano-levels. The modified surfaces used globally in dental clinics are well described and comprehensively reviewed in a review article of this Special Issue [1]. This review article also explores some modified implant surfaces that are highly possible to be clinically used, which are very interesting to the readers investigating biologic interfaces.

In fact, the nature of bone-to-implant contact remains unknown. Whether or not a real bond exists between hard tissues and implants is still under investigation. Although some researchers suggest that the bone-to-implant contact would be a simple physical attachment at the bone–implant interface, Kwon et al. proposed that an actual bond might exist between a bone and an implant surface by showing different shear bond strength values of the grades 2 and 4 commercially pure titanium surfaces that have similar topographies [2].

Although the nature of bone response to an implant surface is still under investigation, various methodological approaches are being developed to enhance the bone healing around the surface. For example, ultraviolet photofunctionalization of the grade 4 commercially pure titanium surface eliminates contaminating hydrocarbon on the surface and highly increases surface hydrophilicity, resulting in the acceleration of osseointegration in vivo, which is shown in an article of this Special Issue [3]. A functional peptide that is involved in cell adhesion is very useful to speed up the bone healing process. This Special Issue contains the evaluation of early bone response to a vitronectin-derived functional peptide-treated sandblasted, large-grit, acid-etched titanium surface [4]. A systematic review of zirconia dental implants describes that the clinical use of implants which are more aesthetic than titanium metal ones will increase [5].

The stable peri-implant soft tissue is another key to the long-term success of dental implants, which is closely associated with the implant-abutment connection structure. Both the soft and hard tissue responses, depending on the structures and abutment material characteristics, are becoming another focused topic in clinical implant dentistry. A review of this Special Issue summarizes the relevant literature to establish guidelines regarding the effects of connection type between abutments and implants in soft and hard tissues [6]. Biomechanical behaviours of implant-abutment connection designs are shown in two articles, and clinical outcomes are presented in one article, depending on the connection designs [7,8,9]. It is necessary for researchers and clinicians to interpret the clinical data in implantology in the light of the old axioms that pocket formation is the initiator for peri-implant or periodontal inflammation and that bone responds to strain, not to stress itself.

Masticatory forces are transferred from superstructures or artificial teeth to bone via implants. A biomechanical model was introduced in the study of Kung et al. for the prediction of bone healing around a dental implant system composed of an artificial crown cemented to a one-body implant, where an abutment and an implant are fused together [10]. Various materials are being developed for superstructures that are usually cemented to abutments. Two major materials are zirconia and glass ceramics, which have been recently supported by digital technology. Interesting mechanical results are shown in an article of this Special Issue, when the zirconia superstructures are cemented or when the superstructures are screw-retained [11]. Intriguingly, Jang et al. evaluated a cemented interface between an artificial crown and an abutment, investigating the effects of cementation methods on the bond strength and fracture resistance between glass–ceramic superstructures and zirconia abutments [12]. In addition, Tribst et al. estimated implant-supported polymer-infiltrated ceramic crowns in vitro when the crowns were cemented to the titanium abutments [13]. These materials and skills were tested in laboratories to reduce the frequent clinical complications of implant-supported superstructures, which are material chipping, crown dislodgement and crown fracture. Long-term studies in clinics designed to evaluate the performances of these materials and skills are being waited for.
